# Turnover intention of hospital staff in Ontario, Canada: exploring the role of frontline supervisors, teamwork, and mindful organizing

**DOI:** 10.1186/s12960-019-0404-2

**Published:** 2019-08-14

**Authors:** Shahram Zaheer, Liane Ginsburg, Hannah J. Wong, Kelly Thomson, Lorna Bain, Zaev Wulffhart

**Affiliations:** 1 School of Health Policy and Management, York University, Toronto, Canada; 2 School of Administrative Studies, York University, Toronto, Canada; 3 Interprofessional Collaboration and Education, Southlake Regional Health Centre, Newmarket, Canada; 4 University of Toronto, Toronto, Canada; 5 Regional Cardiac Care Program, Southlake Regional Health Centre, Newmarket, Canada

**Keywords:** Turnover intention, Teamwork, Supervisory leadership, Mindful organizing, Quality of care

## Abstract

**Background:**

This study contributes to a small but growing body of literature on how context influences employee turnover intention. We examine the impact of staff perceptions of supervisory leadership support for safety, teamwork, and mindful organizing on turnover intention. Interaction effects of safety-specific constructs on turnover intention are also examined.

**Methods:**

Cross-sectional survey data were collected from nurses, allied health professionals, and unit clerks working in intensive care, general medicine, mental health, or the emergency department of a large community hospital in Southern Ontario.

**Results:**

Hierarchical regression analyses showed that staff perceptions of teamwork were significantly associated with turnover intention (*p* < 0.001). Direct associations of supervisory leadership support for safety and mindful organizing with turnover intention were non-significant; however, when staff perceived lower levels of mindful organizing at the frontlines, the positive effect of supervisory leadership on turnover intention was significant (*p* < 0.01).

**Conclusions:**

Our results suggest that, in addition to teamwork perceptions positively affecting turnover intentions, safety-conscious supportive supervisors can help alleviate the negative impact of poor mindful organizing on frontline staff turnover intention. Healthcare organizations should recruit and retain individuals in supervisory roles who prioritize safety and possess adequate relational competencies. They should further dedicate resources to build and strengthen the relational capacities of their supervisory leadership. Moreover, it is important to provide on-site workshops on topics (e.g., conflict management) that can improve the quality of teamwork and consequently reduce employees’ intention to leave their unit/organization.

**Electronic supplementary material:**

The online version of this article (10.1186/s12960-019-0404-2) contains supplementary material, which is available to authorized users.

## Background

### Literature review

Workforce turnover is a normal part of any human resource-based sector and can be beneficial in certain cases, e.g., an organization can select a new employee that is better able to cope with the demands/rigors of a given job. However, turnover is a major cause of concern if it occurs at a high rate in settings already plagued by workforce shortages as is often the case in healthcare systems around the world [1, 2]. High levels of employee turnover have both a direct and indirect negative economic impact on the health sector [1, 3]. Direct costs are tangible and are associated with hiring new employees, e.g., advertising, recruiting costs. Indirect costs, such as decreased initial productivity of new employees and lower group cohesion and morale, while hidden, can be highly problematic for the operational functioning of a unit/organization. Indirect costs are also implicated in creating a “vicious cycle,” whereby increased workload and lower morale of remaining employees increase the likelihood of further turnover [2, 4]. High turnover also negatively effects the well-being of patients. For example, in healthcare settings, high nursing turnover was associated not only with deteriorated nurses’ mental health [5], but also increased rates of resident infections and hospitalization [6] and an increased likelihood of medical errors [5] while lower nurse turnover was associated with decreased rates of medication errors, patient falls, and adverse events [7].

It is often difficult to measure actual turnover rate; consequently, turnover intention is frequently relied upon as a valid proxy for actual leaving behaviors [8] because it is the most immediate and the strongest direct predictor of turnover [1, 9]. Employee’s intent to leave or stay can be defined in terms of unit/department, organization, or occupation [10]. More broadly, turnover intention refers to a conscious and deliberate willingness to leave an organization [11].

### Relational factors affecting turnover intention

Recent literature reviews and meta-analyses suggest that workplace relationships, collaborations, and/or support systems, especially those pertaining to immediate supervisor and coworkers, are important predictors of employee turnover intention in healthcare settings [1, 3, 4, 10, 12]. For example, a qualitative study found that high nursing turnover intention was associated with a variety of interrelated factors including remote and unsupportive management, poor communication, and lack of support from colleagues, i.e., physicians and nurses [13]. Other studies have found that low teamwork scores were associated with higher intention to leave [14] while more support from both supervisors and colleagues was associated with higher intention to stay in public and private healthcare settings [12, 15, 16].

There is a growing realization that healthcare organizations can further improve the quality of care by implementing mindful organizing practices from high reliability organizations (HROs)—e.g., nuclear power plants, air traffic control systems. Mindful organizing practices are characterized by proactive or voluntary extra-role employee behaviors that can help prevent or mitigate incidents capable of jeopardizing the safe functioning of an organization [17, 18], permitting HROs to operate almost error free in highly complex and tightly coupled environments. The beneficial impact of mindful organizing on safety and employee well-being is empirically well established in non-healthcare domains [19]. However, in healthcare, empirical research on mindful organizing is still limited and primarily aimed at understanding its impact on patient safety outcomes. For example, higher mindful organizing at nursing units was shown to result in fewer patient falls and medication errors [20, 21] while violations of mindful organizing at a surgical center led to excessive pediatric cardiac surgical deaths [22]. We are not aware of previous empirical studies that explore the relationship between mindful organizing and turnover intention; however, one prior study exists that has examined the impact of mindful organizing on actual leaving behaviors. Conducted by Vogus and colleagues, the cross-sectional study showed that mindful organizing was associated with significantly lower nursing turnover rates at the unit level in acute care hospitals [23]. Given that employee turnover is less of a concern in traditional HROs compared to healthcare organizations, the dearth of empirical research on how mindful organizing impacts turnover intention and/or actual leaving behaviors might be justified. Nonetheless, as healthcare organizations try to implement HROs’ safety-enhancing concepts such as mindful organizing, it is imperative to further empirically explore the relationships among mindful organizing and other healthcare-relevant contextual factors, including turnover intention and the influence on the quality of care and staff well-being.

### Justification for the current study

The research community has made important inroads in understanding the impact of context-related predictors on turnover intention. However, there are several gaps in the literature on the turnover intention which still need to be addressed. First, past empirical research has primarily focused on certain turnover intention predictors—e.g., job satisfaction—while the impact of other pertinent turnover intention predictors—e.g., mindful organizing—have largely been underexplored. Second, empirical research in healthcare settings has been limited to an examination of the *main effects* of constructs on the outcomes with little attention to potentially important *interactive effects* [24, 25]—there is a need to examine mediating and moderating influences of predictors on turnover intention [1]. Third, empirical research on turnover intention in healthcare settings has primarily focused on understanding the perceptions of nurses while the perspectives of other healthcare professionals remain underexplored. Finally, past research on turnover has suffered from psychometric issues—e.g., use of a single-item turnover intention scale—and conceptual imprecision stemming from the lack of clear definition of turnover intention [8]. Conceptual clarity would also minimize the likelihood of erroneous inclusion of certain predictors (e.g., workload, burnout, retirement, pregnancy, and parental leave) as components of turnover intention in measurement instruments. This would not only strengthen the practical utility of the turnover intention construct but also enable researchers to examine its relationship with related but distinct constructs.

The current study seeks to address the above noted gaps in the turnover intention literature by examining how nurses’, allied health professionals’, and clerical staff’ perceptions of immediate supervisor, teamwork, and mindful organizing impact their turnover intentions. More specifically, it is hypothesized that:

- **Hypothesis 1** *Positive perceptions of supervisory leadership support for safety, teamwork, and mindful organizing will be associated with lower staff turnover intention.*
- **Hypothesis 2** *The predictor variables will interact and significantly influence staff intention to leave.*

## Methods

### Setting

The current study was conducted at a large community hospital 50 km from central Toronto, Canada. The hospital has approximately 400 inpatient beds and offers a variety of speciality services including cancer care, cardiac care, pediatrics, and mental health services.

### Sampling and data collection procedures

Data were obtained from frontline nurses (i.e., registered nurses and registered practical nurses), allied health professionals (AHPs) (e.g., respiratory therapists, physiotherapists, pharmacists), and clerical staff. The study sample included all staff in the above roles who had worked for at least 6 months on one of the four participating clinical units—i.e., intensive care unit (ICU), general medicine, adult inpatient mental health, and emergency department (ED). The exclusion criteria included anyone in a leadership role (e.g., nurse manager) or anyone who was not in direct contact with patients (e.g., clerical staff responsible for the administrative duties such as booking appointments for a nurse manager).

Survey data were collected between September 30, 2015, and February 1, 2016. During that time, the lead author visited each of the four units several times to recruit as many eligible full-time, part-time, and casual staff as possible. Non-probability convenience and snowball sampling procedures were used as it was not feasible to acquire accurate staffing numbers from unit managers since casual staff were supplied by staffing agencies and assigned to a unit based on need. The on-site visits were spread across both the day and night shifts so the researcher could meet and give surveys to as many eligible staff as possible. During each unit visit, a short oral presentation on the study’s purpose, inclusion/exclusion criteria, survey characteristics (e.g., voluntary, anonymous, cross-sectional), etc. were given to solicit staff participation. Surveys were only handed out to the staff that acknowledged that they met the study’s inclusion criteria and were willing to participate in the study. Respondents were asked to indicate the clinical unit they worked on; however, no individual identifiers were solicited (i.e., survey data were anonymous). A drop box was placed on each participating unit to collect completed surveys. As a small incentive to participate, a $20 gift card raffle draw was held on the final day of data collection on each unit. A returned completed survey by a respondent constituted his/her consent to participate in the study.

### Measures

A survey was constructed using previously validated scales to assess participants’ perceptions of supervisory leadership, teamwork, mindful organizing, and turnover intention. Demographic data on tenure, profession, and gender were also collected.

#### Explanatory variables

Supervisory leadership support for safety was measured using the Canadian Patient Safety Climate Survey (Can-PSCS) [26]. The Can-PSCS is a theory-based instrument that has strong psychometric properties validated by confirmatory factor analysis and is currently being used in health settings as part of the Accreditation Canada’s Qmentum Accreditation Program. The supervisory leadership scale reflects the staff perceptions of frontline-level leadership commitment to patient safety. This scale consists of two items (e.g., “my supervisor/manager seriously considers staff suggestions for improving patient safety”) and was previously shown to have strong internal consistency reliability, *α* > 0.80 [26]. Staff perceptions of the quality of teamwork on their respective unit were measured using the Safety Attitudes Questionnaire teamwork climate scale. This scale has six items (e.g., “the physicians and nurses here work together as a well-coordinated team”) and was previously shown to have good psychometric properties (e.g., *α* = 0.78) in acute care settings [27]. The supervisory leadership and teamwork both use a 5-point agreement Likert scale (1 = “disagree strongly” to 5 = “agree strongly”).

The Safety Organizing Scale (SOS) captures the principles of mindful organizing and consists of nine items (e.g., “when errors happen, we discuss how we could have prevented them”), each measured on a 7-point Likert scale (1 = “not at all” to 7 = “to a very great extent”)*. The SOS was previously shown to have good psychometric properties—e.g., α = 0.88* [20].

#### Outcome variable

Turnover intention was operationalized as behavioral intent of an employee to leave his/her current job by either transferring to a different unit in the same organization or by seeking employment at a different organization while staying in his/her occupation. A three-item turnover intention measure was used in this study: “there is a good chance that I will leave this job in the next year or so”; “I frequently think of quitting this job”; and “I will probably look for a new job in the next year.” This turnover intention measure has good psychometric properties and showed good discriminate validity in a confirmatory factor analysis of 45 items on job-related attitudes [28]. Cronbach’s *α* of the scale was previously shown to be > 0.80 [28, 29]. Each item of the turnover intention scale was measured using a 7-point Likert scale where a higher score indicated a higher likelihood that a person would quit his/her current job.

Any negatively phrased items in the supervisory leadership, teamwork, or mindful organizing scales were reverse coded to ensure that a high score on an item corresponded to a high score on a scale. The three negatively phrased items associated with turnover intention scale were not reverse coded as it made intuitive sense that a high score on the scale corresponded to a higher intention to leave. A mean score for each scale was calculated if a respondent answered more than half of the questions associated with that scale. The study survey is provided in Additional file 1.

### Analysis

All analyses were carried out using SPSS, version 11. Manual double entry of survey data was used to minimize data entry errors [30]. Cronbach’s *α* values were calculated for supervisory leadership, teamwork, mindful organizing, and turnover intention to assess the reliability of these scales in the current dataset [31, 32].

Simple bivariate analyses (Pearson *r*) were carried out to assess the strength and significance of the relationships among the dependent and non-demographic independent variables. The residual scatter and probability-probability plots for turnover intention were examined to ensure that the assumptions of multiple linear regression were met [31, 32].

To test our study hypotheses, hierarchical regression analysis was utilized. Hierarchical regression analysis permits a researcher to examine the unique variance accounted for by a predictor, over and above the variance contributed by independent variables entered earlier in an analysis [33]. Demographic variables are typically good candidates for the first step in a hierarchical regression analysis [34], as they are static variables and should be entered in an analysis before the dynamic variables [33]. Hence, unit affiliation and staff demographic (i.e., gender, tenure, and profession) dummy variables were placed in block 1 and block 2 of the hierarchical regression analysis, respectively. The three predictors (i.e., supervisory leadership support for safety, teamwork, and mindful organizing) and their associated interactions were placed in blocks 3 and 4, respectively. All predictors with interactions were centered to avoid problems of multicollinearity [35], and significant interactions were plotted.

## Results

### Response rate and sample characteristics

Table 1 shows the survey response rate for the current study. A total of 245 surveys were distributed. Of these, 185 completed surveys were returned. Two returned surveys were excluded from the study analyses as the respondents indicated that they had worked for < 6 months on their clinical unit. The small number of eligible clinical staff who refused to take a survey was added to the denominator for purposes of calculating the survey response rate.

**Table 1:** Survey response rate by clinical unit

	Distributed	Refused survey at handout	Excluded (ineligible)	Returned	Response rate = returned ÷ (distributed + refused − ineligible)
Intensive care unit	66	2	0	49	49/68 = 72.1%
General medicine	49	0	0	45	45/49 = 91.8%
Emergency department	88	1	1	60	59/88 = 67.0%
Mental health	42	1	1	31	30/42 = 71.4%
Total	245	4	2	185	183/247 = 74.1%

The overall survey response rate was 74.1% (183/247). The survey response rates from the ICU, ED, and mental health were quite similar, ranging from 67% to 72.1% (see Table 1). It is possible that the 91.8% survey response rate on general medicine was facilitated by the physical space constraints of the unit—i.e., the presence of semi-private patient rooms necessitated the charge nurse/unit clerk to ask all the staff to gather for a quick huddle when the primary researcher was on site. These huddles made it easier for the researcher to build a good rapport with the staff and provided participants with an opportunity to complete the survey on the spot. Staff huddles were also conducted at other clinical units to help facilitate data collection, but these occurred less frequently than in the general medicine unit.

Most study participants were female (89.6%) nurses (79.8%) and had a tenure of greater than 5 years on the unit (54.1%). The proportion of nurses (79.8%), AHPs (9.8%), and clerical staff (7.7%) in our survey respondents was similar to their proportion in participating units’ full-time staff where 82.5% were nurses, 9.7% were AHPs, and 7.8% were clerks—see Table 2. Other demographic information for participating clinical units’ full-time nurses, AHPs, and clerical staff were not available.

**Table 2:** Demographic information of the whole sample (*N* = 183)

		Frequency		Percent	
Tenure	6–24 months	24		13.1	
	2–5 years	51		27.9	
	> 5 years	99		54.1	
	No response	9		4.9	
	Total	183		100	
Gender	Female	164		89.6	
	Male	16		8.7	
	No response	3		1.6	
	Total	183		100	
Profession	Nurses	146	*264*	79.8	*82.5*
	Allied health professional (AHP)	18	*31*	9.8	*9.7*
	Clerical staff	14	*25*	7.7	*7.8*
	No response	5	–	2.7	–
	Total	183	*320*	100	*100*

Professional breakdown of full-time staff is reported in italics

### Bivariate analyses

Table 3 shows the results of the bivariate analyses and reveals significant relationships among the predictor and outcome variables with no evidence of multicollinearity. The Cronbach’s *α* value for the teamwork scale was .78, and *α* exceeded .80 for the other scales—alphas are shown in the diagonal in Table 3.

**Table 3:** Means, standard deviations (SD) and Pearson *r* correlations (*N* = 183)

	Mean	SD	1	2	3	4
1. Supervisory leadership	3.61	1.02	*.82*			
2. Teamwork	3.61	.67	.593**	*.78*		
3. Safety Organizing Scale	4.34	.93	.369**	.515**	*.89*	
4. Turnover intention	3.20	1.72	− .140	− .339**	− .234**	*.89*

**p < .01

### Hierarchical linear regression analyses

Table 4 shows the results of the hierarchical regression analyses. The unit demographic variables, when entered in block 1 of the regression model, did not explain a significant amount of variance in turnover intention (ns in Table 4). Similarly, the staff demographic variables, when entered in block 2 of the regression model, did not explain a significant amount of variance in turnover intention (ns in Table 4). However, the *β* coefficients for nurses (*p* < .05) and clerical staff (*p* < .05) were significant, indicating a higher turnover intention for nurses and clerical staff compared to allied health professionals (the reference group).

**Table 4:** Results of hierarchical regression analysis (DV = turnover intention)

	Model 1, *β*	Model 2, *β*	Model 3, *β*	Model 4, *β*
Block 1—Unit affiliation				
ICU	− .397	− .543	− .151	− .146
ED	− .206	− .375	− .278	− .271
Mental health	.648	.590	.390	.473
Block 2—Staff demographics				
Tenure (2–5 years)		.018	− .401	− .376
Tenure (> 5 years)		.537	.156	.119
Female		.087	− .109	− .118
Nurses		1.138*	1.504**	1.554**
Clerical staff		1.415*	1.776**	1.749**
Block 3—Predictor variables				
Supervisory Leadership			.268	.338*
Teamwork			− 1.002***	− 1.097***
Mindful organizing (SOS)			− .229	− .142
Block 4—Interactions				
Supervisory × teamwork				.307
Supervisory × SOS				− .446**
Teamwork × SOS				.358
Total *R*^2^ (adjusted)	.021	.050	.170***	.197*
Change in *R*^2^	.039	.057	.130***	.040*

Reference groups: general medicine, tenure (6–24 months), male, and allied health professionals (*N* = 165)

***p < .001, **p < .01, *p < .05

Supervisory leadership, teamwork, and mindful organizing when entered in block 3 of the regression model explained 13% of variance in turnover intention (*p* < .001), over and above that which was explained by the unit and staff demographic variables entered in previous blocks. The *β* coefficient for teamwork (*p* < .001) was significant.

**Fig. 1:**
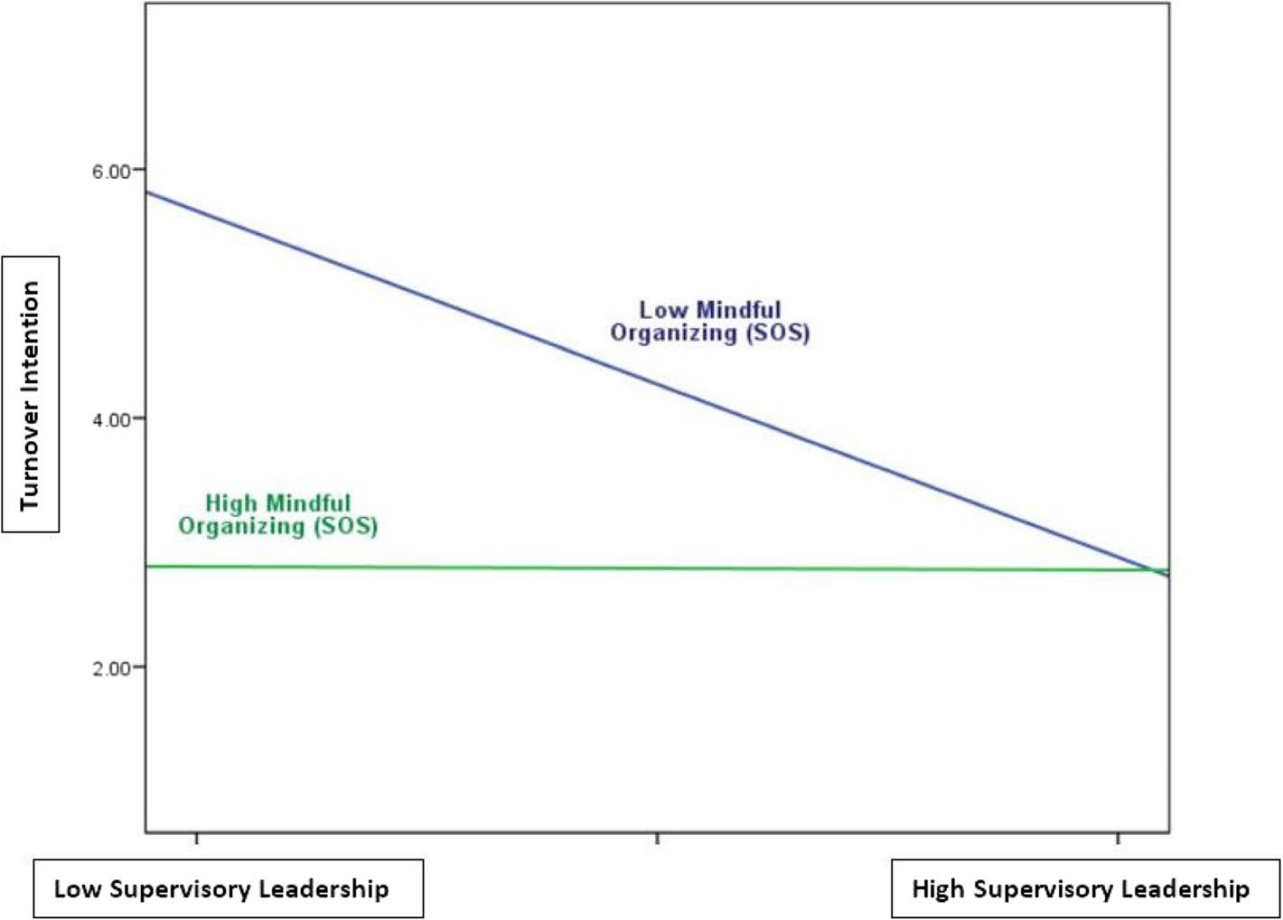
The relationship between supervisory leadership and turnover intention at different levels of mindful organizing (SOS)

Finally, the three interactions, when entered in block 4 of the regression model, explain a significant amount of variance in turnover intention (*p* < .05). However, only the interaction between supervisory leadership and mindful organizing (*p* < .01) was significant. The significant interaction between supervisory leadership and mindful organizing is plotted in Fig. 1. This figure shows that when perceptions of mindful organizing are high, perceptions of supervisory leadership are not associated with turnover intention; however, when perceptions of mindful organizing are low, supervisory leadership becomes an important predictor of turnover intention. In total, the regression model accounted for 20% of the variance in turnover intention.

## Discussion

The survey results only partially supported hypothesis 1 and 2. The direct relationships of supervisory leadership support for safety and mindful organizing with turnover intention were found to be non-significant. Other literature (reviewed above) suggests there is emerging empirical evidence of the positive impact of supportive supervisors on turnover intention. The survey we used solicited staff perceptions of only two *proactive* safety behaviors of a supervisor: (1) encouragement of clinical staff to follow established patient safety procedures and (2) consideration of staff suggestions for improving patient safety. It is possible, even likely, that clinical staff perceive safety-related responsibilities of a supervisor more broadly—e.g., others have suggested that the ability to provide timely feedback for reported errors is seen as a central aspect of supervisory leadership support for safety [36]. Future research that operationalizes supervisory leadership for safety in a broader way may reveal that this variable has a more pronounced direct effect on turnover intention.

In comparison with the current study, all previous empirical research on mindful organizing utilized larger samples which increases the likelihood of detecting significant associations among variables [37]. In addition, high reliability theory may not yet be part of frontline providers’ lexicon to the same extent as other safety-related concepts—e.g., communication, safety culture. Consequently, it is feasible that the current study’s survey respondents were either unaware of, or did not fully appreciate, the importance of extra-role safety behaviors that underpin the safety organizing scale.

Although we did not find evidence of a direct effect of either supervisory leadership or mindful organizing on turnover intention, our results showing a significant interaction between these two predictors make a novel and important contribution to the literature. These findings suggest that supervisory leadership’s positive impact on turnover intention becomes particularly important when staff perceive poorer mindful organizing at the frontlines (see Table 4 and Fig. 1). In other words, a safety-conscious supportive supervisor can compensate when mindful organizing at the frontlines is perceived to be poor and significantly lower staff turnover intention. And as noted, it is possible that a broader operational definition of supervisory leadership would reveal an even more pronounced compensatory effect. To our knowledge, no previous study has empirically examined the interactive impact of supervisory leadership and mindful organizing on turnover intention. This line of enquiry is especially relevant for loosely coupled organizations such as hospitals where frontline managers/supervisors often hold considerable leeway while implementing organizational policies [38, 39].

Our results found that perceptions of teamwork have a significant direct effect on turnover intention—every 1-point increase in teamwork resulted in a 1-point decrease in turnover intention (see Table 4). We also found higher levels of turnover intention among nursing and clerical staff compared to allied health professionals. Certain healthcare professionals—e.g., nurses—are more likely to experience poor quality of teamwork due to a variety of interrelated factors—e.g., power/status hierarchy, lack of autonomy [40]. Others have also found that when healthcare employees perceive a lower quality of teamwork, they are more likely to report higher turnover intention [3] and intention to leave in turn is significantly associated with actual leaving behaviors [9]. Healthcare organizations may be able to reduce nursing and clerical staff turnover by focusing their efforts on improving the quality of teamwork.

### Limitations and future research

This study was cross-sectional, and therefore, causal associations between the predictors and outcome cannot be established. Also, self-reported measures were utilized that are subject to social desirability biases [41]. However, assuring survey participants’ anonymity as was done in the current study likely minimized socially desirable responses [42]. Moreover, while social desirability bias might impact *absolute levels* of teamwork, supervisory leadership, mindful organizing, and turnover intention, it is unlikely to influence the relationships *among* these variables. Common method variance may inflate the magnitude of the relationships we examined as the predictor and outcome variables were taken from the same survey. Our model explains 20% of the variance in turnover intention. Turnover intention may be due to personal (e.g., spousal relocation or maternity leave) or work-related (e.g., job satisfaction) factors. This study only examined the work-related antecedents. Future research should examine the relative influence of personal and work-related factors on turnover intention.

Physicians were not included in the current study since only a small number of full-time physicians worked on general medicine and mental health units. Moreover, physicians are often not physically present on a clinical unit throughout a shift making their recruitment using the study’s data collection procedures difficult. Physicians are also more likely to be informally seen as team leaders by other clinical staff, and the current study did not include clinicians in leadership roles.

Lastly, convenience and snowball sampling procedures were utilized, and data come from a single large community hospital. It is recommended that future research tests the validity of the current study’s inferences in other types of clinical units (e.g., surgery or pediatrics), professions (e.g., physicians), and hospitals (e.g., small community or teaching) using larger multi-site samples.

### Implications for practice

When healthcare employees perceive poor quality of teamwork, they are more likely to report higher turnover intentions as poor teamwork not only hinders their ability to provide good quality care but also negatively impacts their well-being [10]. Therefore, healthcare organizations can provide on-site inter-professional collaborative workshops on topics that can strengthen working relationships including conflict management, negotiation skills, and stress management [43]. In addition, the relational practices—e.g., providing support and constructive feedback—of formal healthcare supervisors which are associated with a lower level of employee turnover intention [10, 12] may also help to foster stronger teamwork climate perceptions. Our results suggest that relational qualities of frontline leaders become particularly important when other aspects of the context, such as perceptions of mindful organizing, are low. Healthcare institutions should focus on recruiting and retaining individuals possessing relational competencies into supervisory leadership roles. In settings where supervisory support for safety is lagging, attention can be directed to a small but growing evidence base that suggests leadership for quality and safety can be built as part of the interventions to improve care [44]. Organizations and health systems are encouraged to view leadership for safety as a modifiable element that can be fostered rather than a fixed aspect of context that is either present or absent [45].

## Conclusion

Healthcare systems around the world are facing employee shortages and high levels of turnover. This problem is especially pronounced in certain healthcare professions such as nursing [4]. The results of the current study lend support to this assertion as nursing and clerical staff had significantly higher turnover intentions compared to the allied health staff. Hence, it is prudent to implement staff retention strategies tailored towards healthcare professions that are more likely to exhibit high turnover intentions. Past research also suggests that increasing recruitment and pay are only short-term solutions while interventions that improve the quality of employees’ work life are more effective long-term solutions to reduce turnover [1]. Indeed, the results of this study show that good perceptions of teamwork significantly lower nursing, allied health, and clerical staff intentions to leave their job. Moreover, when frontline staff perceive poor mindful organizing, a supportive supervisor that prioritizes safety can significantly reduce employees’ turnover intentions. This finding is particularly noteworthy as it highlights the underexplored but important compensatory effect that supportive leadership can have when other aspects of the work context are negative. Together, these results highlight that interventions that improve the quality of teamwork and build/foster supportive supervisory leadership have the potential to lower nursing, allied health, and clerical staff intentions to leave and consequently reduce their actual turnover in the long run.

## Additional file

Additional file 1: Questionnaire items by scale. (DOCX 29 kb)

## Data Availability

The dataset used during the current study is available from the corresponding author, SZ, on reasonable request.

## Abbreviations

AHPs: Allied health professionals
Can-PSCS: Canadian Patient Safety Climate Survey
ED: Emergency department
HROs: High reliability organizations
ICU: Intensive care unit
SOS: Safety Organizing Scale
