# Supplementary material for: Turnover intention of hospital staff in Ontario, Canada: exploring the role of frontline supervisors, teamwork, and mindful organizing
Source: Hum Resour Health. 2019 Aug 14;17:66. doi: 10.1186/s12960-019-0404-2 (PMC6693251; doi:10.1186/s12960-019-0404-2)
Supplement: Supplementary file 1 — Questionnaire items by scale. (DOCX 29 kb) [file 12960_2019_404_MOESM1_ESM.docx]

**Questionnaire items by Scale**

**1.** On which patient care unit you spend the most time? Unit name: ­­­­­­­­­­­­­­­­­­­­__________________________

**2.** For how long have you been working on this unit?

❒ 6-24 months

❒ 2-5 years

❒ > 5 years

**3. Supervisory leadership for safety**

We are looking for your perceptions and opinions of these safety issues. While thinking about your unit, indicate the extent to which you agree or disagree with each of the following statement.

|  | **Strongly Disagree** | **Disagree** | **Neutral** | **Agree** | **Strongly Agree** | **N/A** |
| --- | --- | --- | --- | --- | --- | --- |
| 1. My supervisor/manager says a good word when he/she sees a job done according to established patient safety procedures | ❒ | ❒ | ❒ | ❒ | ❒ | ❒ |
| 1. My supervisor/manager seriously considers staff suggestions for improving patient safety | ❒ | ❒ | ❒ | ❒ | ❒ | ❒ |

**4. Teamwork**

Please think about the unit where you work most when responding to the following statements.

|  | **Strongly Disagree** | **Disagree** | **Neutral** | **Agree** | **Strongly Agree** | **N/A** |
| --- | --- | --- | --- | --- | --- | --- |
| 1. It is easy for personnel in this unit to ask questions when there is something that they do not understand. | ❒ | ❒ | ❒ | ❒ | ❒ | ❒ |
| 1. I have the support I need from other personnel to care for patients. | ❒ | ❒ | ❒ | ❒ | ❒ | ❒ |
| 1. Team input is well received in this unit. | ❒ | ❒ | ❒ | ❒ | ❒ | ❒ |
| 1. In this unit, it is difficult to Speak Up if I perceive a problem with patient care. | ❒ | ❒ | ❒ | ❒ | ❒ | ❒ |
| 1. Disagreements in this unit are resolved appropriately (i.e., not *who* is right, but *what* is best for the patient). | ❒ | ❒ | ❒ | ❒ | ❒ | ❒ |
| 1. The physicians and nurses and other team members here work together as a well-coordinated team. | ❒ | ❒ | ❒ | ❒ | ❒ | ❒ |

**5. Mindful organizing**

Please think about the unit where you work most when responding to the following statements

|  | **Not at all** | **To a very limited extent** | **To a limited extent** | **To a moderate extent** | **To a considerable extent** | **To a great extent** | **To a very great extent** |
| --- | --- | --- | --- | --- | --- | --- | --- |
| 1. We have a good “map” of each other’s talents and skills | ❒ | ❒ | ❒ | ❒ | ❒ | ❒ | ❒ |
| 1. We talk about mistakes and ways to learn from them | ❒ | ❒ | ❒ | ❒ | ❒ | ❒ | ❒ |
| 1. We discuss our unique skills with each other so we know who on the unit has relevant specialized skills and knowledge | ❒ | ❒ | ❒ | ❒ | ❒ | ❒ | ❒ |
| 1. We discuss alternatives as to how to go about our normal work activities | ❒ | ❒ | ❒ | ❒ | ❒ | ❒ | ❒ |
| 1. When giving report to an oncoming nurse/staff, we usually discuss what to look out for | ❒ | ❒ | ❒ | ❒ | ❒ | ❒ | ❒ |
| 1. When attempting to resolve a problem, we take advantage of the unique skills of our colleagues | ❒ | ❒ | ❒ | ❒ | ❒ | ❒ | ❒ |
| 1. We spend time identifying activities we do not want to go wrong | ❒ | ❒ | ❒ | ❒ | ❒ | ❒ | ❒ |
| 1. When errors happen, we discuss how we could have prevented them | ❒ | ❒ | ❒ | ❒ | ❒ | ❒ | ❒ |
| 1. When a patient crisis occurs, we rapidly pool our collective expertise to attempt to resolve it | ❒ | ❒ | ❒ | ❒ | ❒ | ❒ | ❒ |

**5. Turnover intention**

Please think about the unit where you work most when responding to the following statements.

|  | **Strongly Disagree** | **Disagree** | **Somewhat Disagree** | **Neutral** | **Somewhat**  **Agree** | **Agree** | **Strongly Agree** |
| --- | --- | --- | --- | --- | --- | --- | --- |
| 1. There is a good chance that I will leave this job in the next year or so | ❒ | ❒ | ❒ | ❒ | ❒ | ❒ | ❒ |
| 1. I frequently think of quitting this job | ❒ | ❒ | ❒ | ❒ | ❒ | ❒ | ❒ |
| 1. I will probably look for a new job in the next year | ❒ | ❒ | ❒ | ❒ | ❒ | ❒ | ❒ |

**5. Demographic Information**

**Profession:**

**Gender:**

❒Female ❒ Registered Practical Nurse (RPN)

❒ Male ❒ Registered Nurse (RN)

❒ Nurse Practitioner (NP)

❒ Allied Health Professional (AHP)

❒ Clerical Staff

❒ Other: ___________

***– Thank you for completing this questionnaire –***
